# A self-made three-arm clip for closure of a large wound after endoscopic full-thickness resection

**DOI:** 10.1055/a-2248-0284

**Published:** 2024-03-01

**Authors:** Gang Zhao, Furong Ma, Shisheng Cao, Daibao Zhou, Xianxin Liao, Yao Tang, Can Cai

**Affiliations:** 1 629282 Gastroenterology, Wushan County Peopleʼs Hospital of Chongqing, Chongqing, China; 2 585250 Gastroenterology, The Second Affiliated Hospital of Chongqing Medical University, Chongqing, China

Effective wound closure is key for a successful endoscopic full-thickness resection (EFTR). The currently available titanium clips can only be used to close wounds of <2 cm 1 , the over-the-scope (OTS) clip device requires a series of complex operations 2 , and, although the novel through-the-scope twin endoclip (TTS-TC) device is simple and rapid in operation, it is expensive 3 . Based on the principle of the TTS-TC, a three-arm clip (TAC), which is economical, easy to use, and suitable for the closure of large mucosal or submucosal wounds (>3 cm), was made by our team by binding two titanium clips together.

A 68-year-old man was found on gastroscopy to have a submucosal protrusive lesion in the posterior wall of the upper gastric body. Endoscopic ultrasound confirmed that the lesion was originating from the muscularis propria. EFTR was performed to resect the submucosal tumor, leaving a wound of about 3.5 × 3.0 cm. Herein, we present the successful closure of this large wound with our self-made TAC, using the following steps. First, a transparent cap with a groove was created and place on the tip of the endoscope ( Fig. 1 **a** ). Second, two titanium clips were placed inside and outside of the transparent cap ( Fig. 1 **b** ). The two clips were opened, with one jaw of each clip tied together with surgical suture ( Fig. 1 **c** ). The bound clips were accurately placed on either side of the groove in the cap ( Fig. 1 **d** ). After the wound had been fully exposed ( Fig. 2 **a** ), the TAC was delivered to the site of the wound with the help of an assistant ( Fig. 2 **b** ). With the use of this technique, the large wound on the posterior wall of the upper gastric body was successfully turned into two smaller wounds ( Fig. 2 **c–e** ). The decreased wound sizes following use of the TAC meant traditional clips could subsequently be conveniently used to completely close the wound ( Video 1 ).

**Fig. 1:**
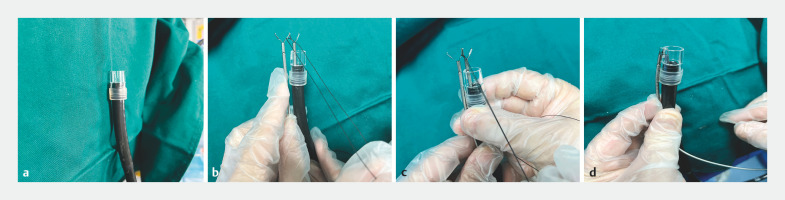
The steps involved in production of a self-made three-arm clip were: **a** a transparent cap with a groove was placed on the tip of the endoscope; **b** a titanium clip passed through the endoscope working channel was positioned inside the cap and a second clip was positioned outside the cap; **c** the two clips were opened, with one jaw of each clip tied together with surgical suture; **d** the two bound clips were accurately placed on either side of the groove in the cap.

**Fig. 2:**
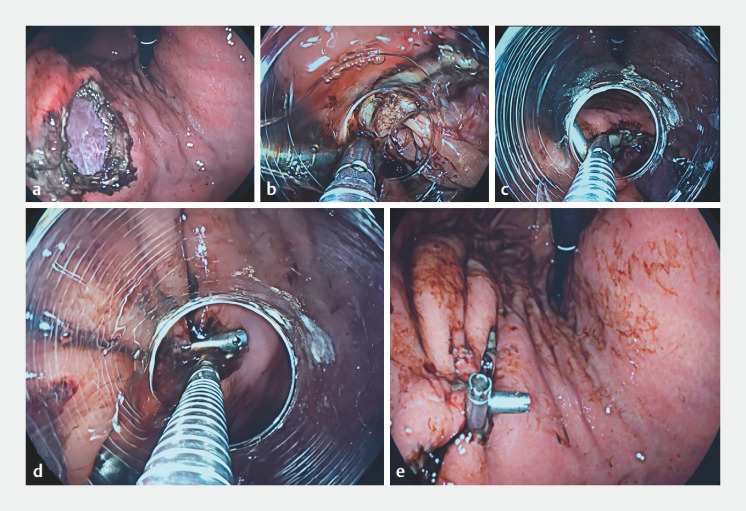
Endoscopic images showing: **a** a large wound on the posterior wall of the upper gastric body following endoscopic full-thickness resection; **b** the self-made three-arm clip (TAC) being delivered to the site of the wound; **c** the tissue on one side of the wound clamped using the outside clip of the TAC; **d** the clamped tissue pulled across to the opposite side of the wound and the inside clip of the TAC used to clamp the tissue on this side; **e** the tissues on either side of the wound clamped together, thereby turning a large wound into two small wounds.

A self-made three-arm clip is used to successfully close a large wound on the posterior wall of the upper gastric body following endoscopic full-thickness resection.Video 1

Endoscopy_UCTN_Code_TTT_1AO_2AI

## Notes

Endoscopy E-Videos https://eref.thieme.de/e-videos
*E-Videos* is an open access online section of the journal *Endoscopy* , reporting on interesting cases and new techniques in gastroenterological endoscopy. All papers include a high-quality video and are published with a Creative Commons CC-BY license. Endoscopy E-Videos qualify for HINARI discounts and waivers and eligibility is automatically checked during the submission process. We grant 100% waivers to articles whose corresponding authors are based in Group A countries and 50% waivers to those who are based in Group B countries as classified by Research4Life (see: https://www.research4life.org/access/eligibility/ ). This section has its own submission website at https://mc.manuscriptcentral.com/e-videos .

## References

[1] Hayashi I Yonezawa TM Kuwabara T The study on staunch clip for the treatment by endoscopy Gastrointest Endosc 1975 17 92 101

[2] Singhal S Changela K Papafragkakis H Over the scope clip: technique and expanding clinical applications J Clin Gastroenterol 2013 47 749 756 10.1097/MCG.0b013e318296ecb9 23751852

[3] Qiang Z Zhen W Yang B A novel through-the-scope twin endoclip for a large mucosal closure in a live pig model Endoscopy 2019 51 E372 E373 31261434 10.1055/a-0948-5252

